# Current smoking with or without chronic bronchitis is independently associated with goblet cell hyperplasia in healthy smokers and COPD subjects

**DOI:** 10.1038/s41598-020-77229-1

**Published:** 2020-11-18

**Authors:** Victor Kim, Stephanie Jeong, Huaqing Zhao, Mehmet Kesimer, Richard C. Boucher, J. Michael Wells, Stephanie A. Christenson, MeiLan K. Han, Mark Dransfield, Robert Paine, Christopher B. Cooper, Igor Barjaktarevic, Russell Bowler, Jeffrey L. Curtis, Robert J. Kaner, Sarah L. O’Beirne, Wanda K. O’Neal, Stephen I. Rennard, Fernando J. Martinez, Prescott G. Woodruff

**Affiliations:** 1 Lewis Katz School of Medicine at Temple University, 3401 North Broad Street, 785 Parkinson Pavilion, Philadelphia, PA 19140 USA; 2 University of North Carolina School of Medicine, Chapel Hill, NC USA; 3 University of Alabama at Birmingham, Birmingham, AL USA; 4 University of California San Francisco, San Francisco, CA USA; 5 University of Michigan School of Medicine, Ann Arbor, MI USA; 6 University of Utah Health, Salt Lake City, UT USA; 7 David Geffen School of Medicine, University of California, Los Angeles, CA USA; 8 National Jewish Health, Denver, CO USA; 9 University of Michigan Health Care System, Ann Arbor, MI USA; 10 Weill Cornell Medical College, New York, NY USA; 11 University of Nebraska Medical Center, Omaha, NE USA

**Keywords:** Chronic obstructive pulmonary disease, Translational research

## Abstract

COPD, chronic bronchitis (CB) and active smoking have all been associated with goblet cell hyperplasia (GCH) in small studies. Active smoking is strongly associated with CB, but there is a disconnect between CB clinical symptoms and pathology. Chronic cough and sputum production poorly correlate with the presence of GCH or COPD. We hypothesized that the primary determinant of GCH in ever smokers with or without airflow obstruction is active smoking. Goblet Cell Density (GCD) was measured in 71 current or former smokers [32 subjects without COPD and 39 COPD subjects]. Endobronchial mucosal biopsies were stained with Periodic Acid Schiff-Alcian Blue, and GCD was measured as number of goblet cells/mm basement membrane. GCD was divided into tertiles based on log_10_ transformed values. Log_10_GCD was greater in current smokers compared to former smokers. Those with classically defined CB or SGRQ defined CB had a greater log_10_ GCD compared to those without CB. Current smoking was independently associated with tertile 3 (high log_10_GCD) whereas CB was not in multivariable regression when adjusting for lung function and demographics. These results suggest that GCH is induced by active smoke exposure and does not necessarily correlate with the clinical symptoms of CB.

## Introduction

Chronic Obstructive Pulmonary Disease (COPD) is a major cause of morbidity and is the fourth leading cause of death in the United States^1^. Chronic bronchitis (CB) is a common phenomenon in smokers with and without COPD and is characterized by chronic cough and phlegm. CB increases risk of respiratory exacerbations, is associated with higher mortality, and hastens lung function decline^2^. The most well-established risk factor for developing CB is active smoking^3,4^.

The pathologic correlate of CB is thought to be goblet cell hyperplasia (GCH). Small airway mucus plugging is more commonly seen in COPD and increases as the degree of airflow obstruction worsens^5^. GCH has been shown to involve the peripheral airways in surgical lung specimens from those with CB^6^ and in large airway endobronchial mucosal biopsies in smokers with airflow obstruction^7,8^. We have previously shown that GCH was greater in the large airways in those with CB compared to those without CB^8^. Additionally, mucus burden has prognostic significance; one study in lung volume reduction surgery patients found that small airway mucous metaplasia inversely correlated with changes in lung function after surgery^9^, whereas another study found that the degree of small airway mucus luminal occlusion correlated with mortality^10^.

However, there is a disconnect between respiratory symptoms and the magnitude of GCH. Although active smoking is the primary risk factor for CB, not all smokers develop CB, and CB can affect former smokers as well^3^. One study in advanced emphysema patients found no relationship between cough and sputum symptoms and degree of small airway mucus impaction^11^, while an established pathologic measure of mucous gland hyperplasia has little to no correlation with clinical symptoms^12^. We sought to analyze GCH as it related to smoking status and chronic bronchitis in smokers with and without airflow obstruction. Given the disconnect between the clinical syndrome of CB and airway pathology, we hypothesized that GCH would be more commonly seen in current smokers compared to ex-smokers and would not necessarily associate with CB.

## Materials and methods

The Subpopulations Intermediate Outcome Measures in COPD Study (SPIROMICS) is a prospective cohort study that has enrolled 2981 subjects across four strata (1) Never smokers (NS), (2) Current or former smokers without COPD (HS), (3) Mild/Moderate COPD, and (4) Severe COPD). Goblet Cell Density (GCD) was measured in 71 subjects in strata 2–4 (current or former smokers with and without COPD) in which endobronchial mucosal biopsies were obtained during the SPIROMICS bronchoscopy substudy^13^. Methods of measuring GCD were performed as previously described^8^. Briefly, biopsies were stained with Periodic Acid Schiff-Alcian Blue. Goblet cells from 4–6 endobronchial specimens were counted and related to the length of basement membrane using Image J^14^. If there were fewer than 4 acceptable samples, the subject was excluded (n = 28 out of 99 total subjects). GCD was expressed as the number of goblet cells per millimeter of basement membrane. Two observers performed the measurements in a double-blinded fashion. GCD was then log_10_ transformed to make the distribution of values assume a more Gaussian distribution and were divided into tertiles (1 = low, 2 = medium, 3 = high GCD). Ethical approval for this study was obtained for this study from the SPIROMICS Observational Safety Monitoring Board and this study was carried out in accordance with the Declaration of Helsinki. All participants were enrolled with informed consent in accordance with and under the approval of the Institutional Review Board (IRB) of participating sites (Columbia University Medical Center- Division of General Medicine, New York, NY; Wake Forest Baptist Health- Center for Genomics and Personalized Medicine Research, Winston Salem, NC; University of Utah- Pulmonary Lung Health Research Center, Salt Lake City, UT; University Of Michigan- Pulmonary and Critical Care Medicine, Ann Arbor, MI; University of California at Los Angeles- Pulmonary and Critical Care Medicine, Los Angeles, CA; University of California at San Francisco- Airway Clinical Research Center; San Francisco, CA; University of North Carolina at Chapel Hill—Genomics and Informatics Center—Collaborative Studies Coordinating Center, Chapel Hill, NC; National Jewish Health- Division of Pulmonary, Critical Care, and Sleep Medicine, Denver, CO; Johns Hopkins University- Pulmonary and Critical Care Medicine, Baltimore, MD; University of Illinois- Breathe Chicago Center, Chicago, IL; University of Alabama at Birmingham- Lung Health Center, Birmingham, AL; Temple University- Dept. of Thoracic Medicine and Surgery, Philadelphia, PA; University of Iowa- Internal Medicine, Iowa City, IA).

Airway total mucin concentration was measured in induced sputum samples in a subset of subjects (n = 7 in tertile 1, n = 15 in tertile 2, n = 6 in tertile 3) using previously described methods^15^. Sputum was induced by inhalation of hypertonic saline by subjects who had a forced expiratory volume in 1 s of more than 35% of the predicted value, according to protocol^16^ and American Thoracic Society and European Respiratory Society standards^17^.

### Statistics

Statistics were performed using SPSS v25 (IBM Corp., Armonk, NY). Intraclass correlation coefficients between the two observers for goblet cells and GCD were calculated. Differences between groups were assessed using either unpaired t tests or one way ANOVA for continuous variables and chi squared tests for categorical variables. Multivariable logistic regression was performed with Tertile 3 (High GCD) as the dependent variable of interest with current smoking, CB classic definition and CB SGRQ definition in separate models with demographics and FEV_1_% predicted as covariates. Additionally, multivariable linear regression was performed with the same covariates for log_10_ GCD. A p value of less than 0.05 was considered statistically significant.

### Ethics approval and consent to participate

Each site had IRB approval and all subjects provided informed consent to participate.

### Consent for publication

All authors provide their consent to participate. There are no individual subject data that are within this manuscript.

## Results

**Figure 1:**
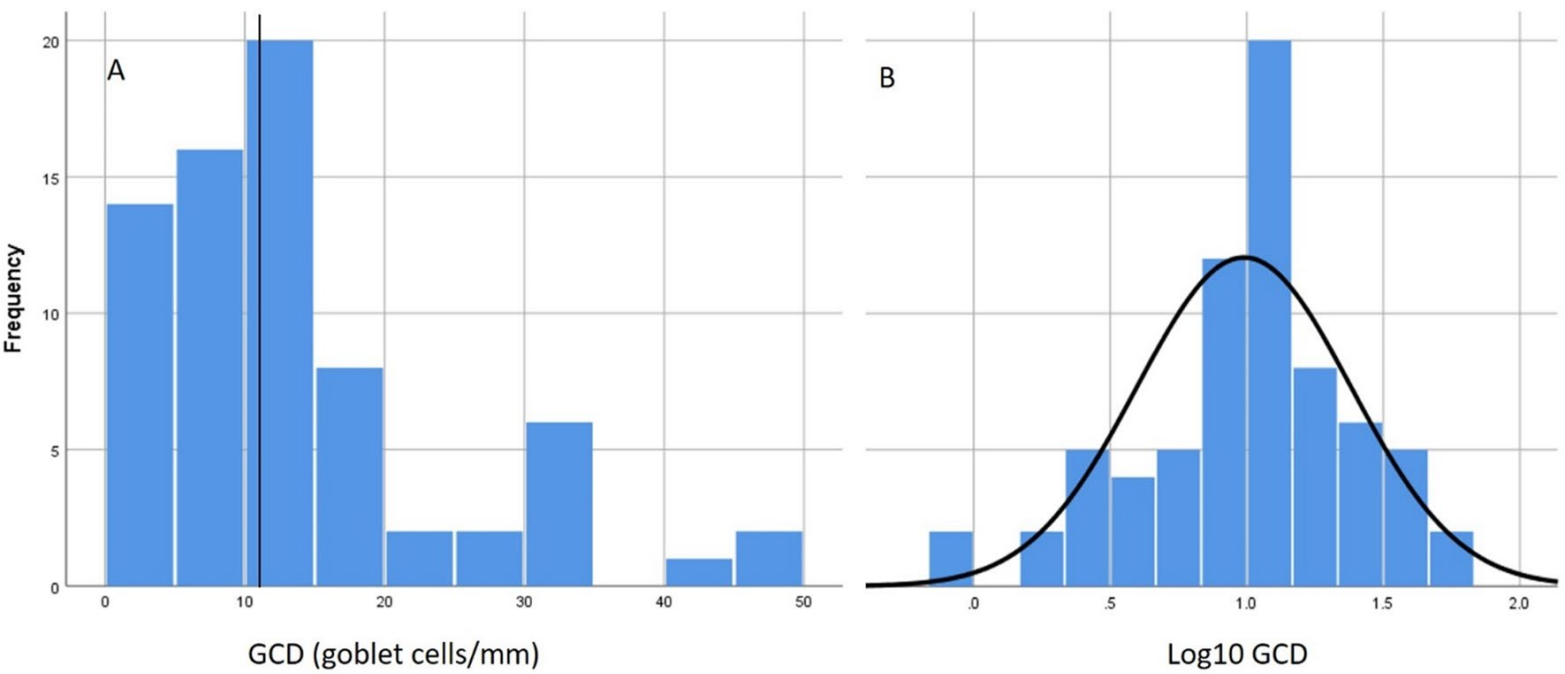
(**A**) Distribution of goblet cell density measurements. Vertical line represents median value. (**B**) Distribution of log_10_ goblet cell density measurements.

**Figure 2:**
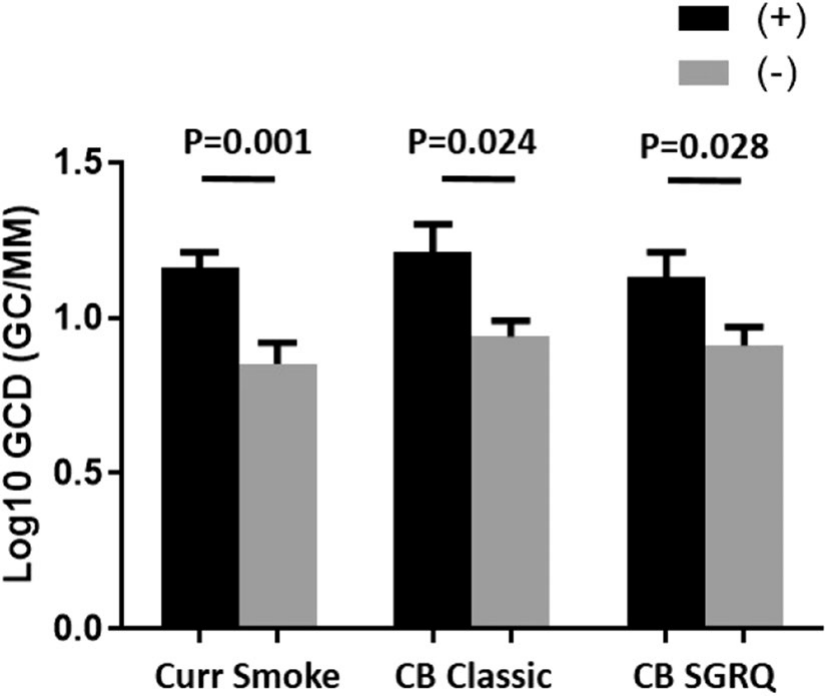
Log_10_ goblet cell density by chronic bronchitis or smoking groups. Data expressed as mean ± SE. *GCD* goblet cell density, *CB* chronic bronchitis, *SGRQ* Saint George’s respiratory questionnaire.

Intraclass correlation coefficients between the two observers for goblet cells and GCD were 0.932 and 0.968, respectively (p < 0.0001 for both). See Fig. 1a for a histogram of the distribution of GCD. Figure 1b shows the distribution of log_10_ GCD. The median value for GCD was 11.034 GC/mm. Log_10_ GCD was greater in those that were currently smoking compared to former smokers (1.16 ± 0.28 [n = 31] vs. 0.85 ± 0.42 [n = 40], p = 0.001). Those with CB defined using the classic definition (cough and phlegm for > 3 months/year for at least 2 consecutive years) had a greater log_10_ GCD compared to those without CB (1.21 ± 0.31 [n = 13] vs. 0.94 ± 0.39 [n = 58], p = 0.024). Similarly, those with CB by the SGRQ definition (cough and phlegm almost every day or several days a week for the past 4 weeks) had a greater log_10_ GCD compared to those without CB (1.13 ± 0.39 [n = 25] vs. 0.91 ± 0.39 [n = 43], p = 0.028). See Fig. 2. Sputum mucin concentration was neither different between those with classic CB compared to those without classic CB (2980 ± 1850 vs. 1724 ± 1279 µg/mL, p = 0.061) nor different between those with SGRQ CB compared to those without SGRQ CB (2090 ± 1688 vs. 1919 ± 1370 µg/mL, p = 0.767).

**Figure 3:**
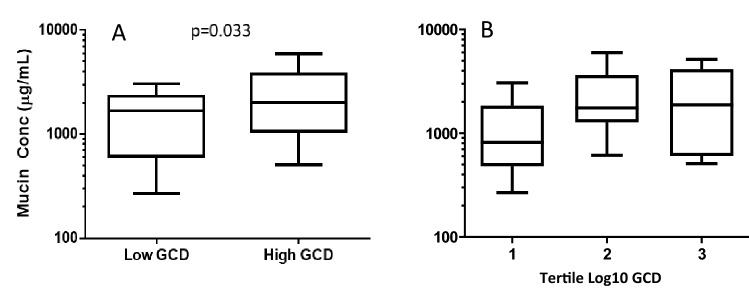
Induced sputum mucin concentrations in the (**A**) low and high GCD groups and the (**B**) three tertiles of log_10_GCD.

The characteristics of the three tertiles are shown in Table 1. 5.0% of ex-smokers and 33.3% of current smokers had classically defined CB. Tertiles 2 and 3 tended to be younger than tertile 1 (64.9 ± 7.5, 59.8 ± 9.3, 59.8 ± 8.2 years in tertiles 1, 2 and 3, respectively, p = 0.06). The groups were similar in gender, race distribution and body mass index. 16.7% of those in tertile 1 were current smokers, whereas in tertiles 2 and 3 the percentages of those that were current smokers were 50.0% and 63.6%, respectively (p = 0.004). The percentages of subjects in each tertile with Classic CB or SGRQ CB were not significantly different. Lung function tended to be lower in tertiles 2 and 3 compared to tertile 1 (FEV_1_ 95.9 ± 20.8, 84.1 ± 16.4, 85.4 ± 19.6% predicted for tertiles 1, 2 and 3, respectively, p = 0.069). 6-min walk distance, SGRQ scores, mMRC dyspnea scores and exacerbation histories were similar between tertiles. CAT scores tended to be worse in tertiles 2 and 3 compared to tertile 1 (7.45 ± 7.58, 12.52 ± 8.46, 11.83 ± 7.95 for tertiles 1, 2, and 3, respectively, p = 0.08). Mucin concentrations were greater in tertiles 2 and 3 but the differences were not statistically significant (1185 ± 967, 2211 ± 1482, 2312 ± 1915 µg/mL in tertiles 1, 2 and 3, respectively, p = 0.279). When GCD was divided into two groups by the median value into Low GCD and High GCD, mucin concentrations were higher in the High GCD group (2492 ± 1691 vs. 1289 ± 838 µg/mL, p = 0.033). See Fig. 3.

**Table 1:** Baseline characteristics.

	Tertile 1 (n = 24)		Tertile 2 (n = 24)		Tertile 3 (n = 23)	
Age (years)	64.9	± 7.5	59.8	± 9.3	59.8	± 8.2
Gender (% male)	13 (54.2)		13 (54.2)		16 (69.6)	
Race (% Caucasian)	20 (83.3)		16 (66.7)		16 (69.6)	
BMI (kg/m^2^)	29.1	± 4.6	28.4	± 4.5	26.9	± 5.7
Current smoker [n (%)]*	4 (16.7)		12 (50.0)		14 (63.6)	
Pack year history	45.2	± 19.8	48.3	± 25.7	43.5	± 20.6
CB classic def [n (%)]	2 (8.3)		4 (16.7)		7 (30.4)	
CB SGRQ def [n (%)]	5 (22.7)		8 (34.8)		12 (52.2)	
FEV1%Pred	95.9	± 20.8	84.1	± 16.4	85.4	± 19.6
FEV1/FVC (%pred)	92.2	± 15.5	83.8	± 14.7	84.0	± 16.7
No COPD [n(%)]	15 (62.5)		7 (29.2)		10 (43.5)	
COPD [n(%)]	9 (37.5)		17 (70.8)		13 (56.5)	
6MWD (m)	477	± 92	439	± 88	466	± 66
SGRQ score	16.1	± 17.3	26.1	± 17.6	23.7	± 19.1
mMRC dyspnea score	0.30	± 0.64	0.65	± 0.89	0.61	± 0.72
CAT score	7.45	± 7.58	12.52	± 8.46	11.83	± 7.95
Exac hx (exac.pt/year)	0.08	± 0.28	0.54	± 1.18	0.41	± 0.80
Sev Exac Hx (exac.pt/year)	0.04	± 0.20	0.17	± 0.38	0.09	± 0.43
GCD (cells/mm)†	4.30	± 2.18	11.20	± 2.00	26.06	± 10.59
Mucin conc (µg/mL)	1185	± 967	2211	± 1482	2312	± 1915

*BMI* body mass index, *CB* chronic bronchitis, *SGRQ* Saint George’s Respiratory Questionnaire, *FEV*_*1*_ forced expiratory volume in 1 s, *FVC* forced vital capacity, *6MWD* 6-min walk distance, *mMRC* modified medical research council, *CAT* COPD assessment test, *Exac Hx* exacerbation history, *Sev Exac Hx* severe exacerbation history, *GCD* goblet cell density.

*p = 0.004, ^†^p < 0.0001.

Multivariable logistic regressions for tertile 3 (high log_10_ GCD) using current smoking, Classic CB and SGRQ CB in separate models are shown in Table 2. Current smoking was independently associated with High GCD after adjusting for other covariates (OR 4.15, 95% CI 1.17, 14.71). Neither Classic CB nor SGRQ CB were associated with High GCD (OR 3.14, 95% CI 0.86, 11.51 and OR 2.42, 95% CI 0.77, 7.56, respectively). Multivariable linear regressions for log_10_ GCD using current smoking, Classic CB and SGRQ CB in separate models are shown in Table 3. Model one uses current smoking, Classic CB or SGRQ CB as independent variables of interest with age, gender and race as covariates. Model two adjusts for age, gender, race, and FEV_1_%predicted. Current smoking and Classic CB were independently associated with log_10_ GCD in model one (estimate 0.265, SE 0.103, p = 0.012 and estimate 0.257, SE 0.115, p = 0.029, respectively). However, in model two, only current smoking was independently associated with log_10_ GCD (estimate 0.226, SE 0.100, p = 0.028).

**Table 2:** Multivariable logistic regression for Tertile 3 (high GCD).

	OR	95% CI	
Current smoking	4.15	1.17	14.71
CB classic def	3.14	0.86	11.51
CB SGRQ def	2.42	0.77	7.56

Covariates include age, gender, race, and FEV_1_%predicted. Current smoking, CB classic definition and CB SGRQ definition were in separate models.

**Table 3:** Multivariable linear regression for log_10_ GCD.

	Model 1			Model 2		
	Estimate	S.E	p	Estimate	S.E	p
Current smoking	0.265	0.103	**0.012**	0.226	0.100	**0.028**
CB classic def	0.257	0.115	**0.029**	0.218	0.111	0.054
CB SGRQ def	0.190	0.098	0.058	0.118	0.100	0.240

Model one is adjusted for age, gender, and race; model two is adjusted for age, gender, race, and FEV_1_%predicted. Current smoking, CB classic definition and CB SGRQ definition were each tested in separate models with the covariates.

The bolded p values are considered statistically significant.

## Discussion

We showed that GCD was greater in those that were currently smoking and in those with CB. Similarly, those with high log_10_ GCD were more likely to be current smokers but not CB using either definition. Using multivariable logistic regression, we showed that current smoking, but not CB, was independently associated with high log_10_ GCD. Similar results were seen with multivariable linear regression after adjusting for lung function and demographics. These results suggest that GCH is induced by active smoke exposure and does not necessarily translate into the clinical symptoms of CB.

This is the first study that demonstrates the complex interrelationship between CB, smoking, and GCH in a large cohort of extensively characterized smokers with and without airflow obstruction. A prior study of epithelial mucin stores compared goblet cell measures in 24 active smokers and 19 nonsmoking controls; mucin stores were greater in the smokers, especially those with airflow limitation^7^. However, it was not clear whether the smokers had CB. In a prior report, we contrasted the GCD in 15 subjects with moderate to severe COPD, 12 smokers without COPD, and 11 healthy nonsmokers^8^. Interestingly, we found that the smokers without airflow obstruction had the greatest degree of GCH while those with CB had a greater GCD than those without CB^8^. Another study revealed that GCH, assessed using qualitative measures, was more commonly seen in habitual tobacco smokers compared to nonsmoking controls^18^. Our data expand upon these findings in a much larger cohort with the addition of the relationship of current smoking with CB.

We also showed that mucin concentrations in induced sputum were greater in those with high GCD. This is the first study that has shown a relationship between large airway GCH and sputum mucins. It has been speculated that mucus is expectorated from large airways to produce sputum, suggesting sputum mucin concentration and large airway GCH should be related, but few studies have addressed the correspondence between these two metrics of mucus production. This association may have clinical relevance as well, as sputum mucins have been related to CB, respiratory exacerbations and peripheral airway disease in 917 subjects in the SPIROMICS cohort^15,19^. Unlike this larger study, our analysis did not show a statistically greater degree of sputum mucins in chronic bronchitics. This may be due to the small sample size or that some mucins arise from submucosal glands that we were not able to examine.

The prevalence of CB ranges anywhere from 12.2% in smokers without airflow obstruction to 74% in some COPD cohorts^20–22^. CB has been related to an accelerated rate of lung function decline, worse health related quality of life, increased mortality, and an increased risk of respiratory exacerbations in individuals with and without COPD^20,23–25^. In the Copenhagen City Heart Study, chronic mucus hypersecretion was associated with an increased rate of FEV_1_ decline over time^24^. Analyses of the COPDGene study and SPIROMICS showed that CB in those without airflow obstruction was associated with respiratory exacerbations^20,26^, and in those with airflow obstruction CB was associated with a two-fold increased rate of exacerbations in longitudinal follow-up^27^. In the Tucson Epidemiologic Survey of Airway Obstructive Disease, younger patients with CB had a greater mortality compared to those without CB^28^. In a study of nearly 48,000 men and women, CB was associated with an increased duration of hospitalization and a greater all-cause mortality^25^.

The most well described risk factor for CB is active smoking. A large study of more than 1,700 Finnish men showed that over 30 years the cumulative incidence of CB in continued smokers was 42%^29^. A meta-analysis revealed that the relative risk of CB from current smoking was 3.41^30^. There is also evidence that smoking cessation decreases CB. Over five years, an analysis of the COPDGene study showed that ex-smokers who resumed smoking were more likely to develop CB and that current smokers who quit were more likely to have their CB resolve^31^. A larger study of over 4,000 subjects followed from ages 20 to 64 showed that those who smoked were more likely to develop chronic mucus hypersecretion and quitting smoking resulted in decreased chronic mucus hypersecretion^4^.

GCH is one of the pathologic foundations for CB. The primary mechanisms responsible for excessive mucus in CB are overproduction and hypersecretion by goblet cells and/or decreased clearance of mucus. Mucus hypersecretion develops as a consequence of cigarette smoke exposure^32,33^, acute and chronic viral infection^34^ and inflammatory cells may activate mucin gene transcription^35^. The increased epithelial gene expression is associated with metaplastic responses in club cells to assume a goblet cell morphology characterized by overproduction of mucus and hypersecretion associated with increased degranulation. This hypersecretion is compounded by difficulty in clearing secretions because of poor ciliary function, defective mucus hydration, distal airway occlusion, and ineffective cough secondary to respiratory muscle weakness and reduced peak expiratory flow in COPD^5,35–37^.

Unlike the tight correlation between emphysema and lung function, the relationship between airway pathology, physiology and symptom severity is only moderate at best. Large airway GCH correlates poorly with the degree of airflow obstruction^38^ or chronic phlegm^39^. Small airway disease has been found in surgical lung specimens from those with advanced emphysema, with no clinical or radiographic evidence to suggest its presence preoperatively^5,9,40,41^. More importantly, the degree of small airway GCH is difficult to detect clinically by cough or sputum burden^11^. Our findings shed light on the subject and improve our current understanding. Although both active smokers and those with CB had increased GCD, only current smoking was independently associated with it in multivariable analysis.

There are several limitations that are worthy of mention. Firstly, although the size of the cohort analyzed is large for a bronchoscopy study, its size in comparison to the entire SPIROMICS cohort is small. Secondly, mucin concentrations on the induced sputum samples were only available for 28 subjects, making the distinction of mucin concentrations between groups suboptimal. There was also a lack of never smokers to serve as a control group. Lastly, there may have been within subject differences in GCD based on the areas sampled which could not be assessed.

Nonetheless, we have shown that goblet cell hyperplasia is related more so to current smoking as opposed to the presence or absence of chronic bronchitis, no matter how it is defined. These findings suggest that goblet cell hyperplasia may exist in the absence of chronic bronchitis, again emphasizing the disconnect between clinical symptoms and airway pathology. However, our findings also suggest that current smoking may cause goblet cell hyperplasia before chronic bronchitis develops or independently from chronic bronchitis. These findings need to be validated in other studies and their clinical relevance need to be better defined.

## Data Availability

More information about the study and how to access SPIROMICS data is at www.spiromics.org.

## Abbreviations

ANOVA: Analysis of variance
ATS: American thoracic society
BMI: Body mass index
CB: Chronic bronchitis
COPD: Chronic obstructive pulmonary disease
CT: Computed tomography
FEV_1_: Forced expiratory volume in 1 s
FVC: Forced vital capacity
GCD: Goblet cell density
GCH: Goblet cell hyperplasia
GOLD: Global initiative for chronic obstructive lung disease
mMRC: Modified medical research council
SGRQ: Saint George’s respiratory questionnaire
SPIROMICS: Subpopulations and intermediate outcome measures in COPD study
